# A Method for Detecting Cattle Behaviors Based on RGB-Depth Dual-Modal Information Fusion

**DOI:** 10.3390/ani16142259

**Published:** 2026-07-21

**Authors:** Zihao Chen, Jiaxing Xie, Liang Mao, Qiuxia Chen, Linlin Wang

**Affiliations:** 1 School of Artificial Intelligence, Shenzhen Polytechnic University, Shenzhen 518055, China; czh_@stu.scau.edu.cn (Z.C.);; 2 College of Artificial Intelligence and Low-Altitude Technology, South China Agricultural University, Guangzhou 510642, China; xjx1998@scau.edu.cn

**Keywords:** cattle behavior, dual-modal, target detection, intelligent ranch

## Abstract

In large-scale cattle farming, accurate behavior recognition is central to achieving intensive health monitoring and animal welfare assessment. To address challenges such as background interference from fences, feed troughs, and stains in real-world barns, and overlapping of cattle coupled with the inability of single-RGB modalities to capture physical spatial structure, which leads to issues like blurred detection boundaries and significant noise interference—we propose a cattle behavior detection method based on RGB-Depth dual-modal information fusion. This approach jointly models the texture information from RGB images and the spatial structural information from depth images. Within this framework, this paper constructs three collaborative optimization modules: first, the CDSAM module is developed, which evaluates neuron importance through a parameter-free attention mechanism and combines dynamic convolutions to adapt to the cattle’s variable postures, effectively suppressing complex background noise. Second, we propose the C2BRA module based on a two-layer routed attention mechanism. By adopting a two-stage modeling approach of “region-level routing—intra-region fine-grained attention,” it adapts to changes in target scale and enhances the model’s ability to represent spatial context for multi-scale semantic information. Finally, in the prediction stage, a lightweight shared convolutional detection head (LSCD) is introduced. By sharing convolutional parameters across scales and decoupling the classification and regression architectures, it reduces computational overhead while maintaining accuracy. Experimental results show that the improved model achieves a mAP@0.5 of 90.3% on our self-built cattle behavior dataset, representing a 4.3 percentage point increase compared to the baseline model, while reducing GFLOPs from 11.0 G to 9.6 G, a decrease of 12.7%; Visualization results indicate that the improved model can focus more accurately on cattle body contours and key behavioral regions, thereby reducing false negatives and enhancing detection accuracy. Concurrently, the model achieves an optimal balance between detection performance and computational complexity, providing robust technical support for automated cattle behavior monitoring on smart farms.

## Simple Summary

In real-world livestock farming scenarios, automatic behavior recognition in cattle often lacks accuracy due to limited behavioral data and background interference. This study proposes a new, more compact model, DMNet, to address this issue. The model effectively utilizes limited dual-modal data to capture more representative behavioral information from images while reducing computational overhead. Additionally, the newly designed recognition architecture enhances noise suppression capabilities and achieves higher accuracy in complex environments. Experimental results demonstrate that DMNet achieves a mAP@0.5 of 90.3% while maintaining extremely low computational requirements, outperforming existing baseline models.

## 1. Introduction

Animal behavior recognition systems have been widely adopted in fields such as ecological research, animal welfare assessment, and smart livestock farming. With the rapid advancement of computer vision and artificial intelligence technologies, and the emergence of intelligent IoT systems and AI-enabled edge computing [1], automated behavior analysis based on images and videos has gradually become an important technical means for animal behavior research. Relevant studies indicate that behavioral patterns exhibited by animals during daily activities—such as standing, lying down, feeding, walking, and social interactions—are closely related to their health status, physiological condition, and production performance. Even subtle differences in behavioral frequency, duration, or postural changes may reflect potential risks such as disease, stress, or environmental discomfort, thereby holding significant practical value and scientific significance in herd management, health early warning, and precision intervention [2,3].

As a major economic livestock species in modern animal husbandry, the behavioral state of cattle directly impacts their growth performance, reproductive efficiency, and welfare levels. In large-scale and intensive farming settings, how to conduct long-term, continuous, and precise automated monitoring of cattle behavior has become a key issue in the development of smart farms. In practical production, behavioral recognition technology provides crucial evidence for herd health assessment, precision feeding, reproductive management, and early warning of abnormal behaviors, thereby enhancing the scientific and intelligent levels of livestock management [4].

Existing cattle behavior monitoring methods are primarily divided into two categories: contact-based sensor methods and non-contact visual analysis methods [5,6]. Contact-based methods typically involve attaching accelerometers, gyroscopes, or rumination sensors to locations such as the cattle’s neck, legs, or ear tags to collect movement data. Dutta R et al. [7] used neck sensors to collect cattle movement data and introduced ensemble learning methods into the automatic recognition of dairy cow behavior, achieving dynamic classification of multiple behavior types. Rahman A et al. [8] systematically evaluated the behavioral recognition potential of three common sensor locations—harness, collar, and ear tag—and used a lightweight random forest classifier to identify six behavioral categories (grazing, feeding, rumination, walking, standing, and lying down) on a per-second basis. Peng Y et al. [9] based on an inertial measurement unit (IMU) containing sensors such as accelerometers, applied a Long Short-Term Memory (LSTM) network for the first time to the fine-grained behavioral recognition of dairy cows, performing end-to-end classification of six daily behaviors—walking, standing, feeding, ruminating, lying down, and scratching—significantly improving the accuracy and robustness of sensor-based behavioral recognition. Although sensor-based approaches offer advantages such as high adaptability and low susceptibility to environmental factors, they suffer from poor wear compliance, high maintenance costs, and the risk of behavioral interference. Furthermore, they struggle to capture spatial relationships among cattle and group behavioral patterns, resulting in significant limitations for long-term application in large-scale real-world scenarios.

In contrast, non-contact methods rely on computer vision and deep learning models to analyze and recognize animal behavior [10,11,12]. Qiao et al. [13] proposed the YOLOv5-ASFF object detection model, which achieves detection of individual cattle, heads, and legs with an accuracy of 96.2%. Bai et al. [14] constructed a Res2 backbone network to integrate multi-scale receptive fields, thereby enhancing YOLOv3’s ability to extract multi-scale features in cattle behavior scenarios. Tong et al. [15] proposed the YOLO-BoT algorithm to achieve high-precision cattle behavior recognition and multi-object tracking in complex pasture environments. Ni et al. [16] proposed IMTFF-Networks, a deep learning model that fuses multimodal spatio-temporal features, to improve the classification accuracy of key cattle behaviors such as rumination and feeding. Overall, deep learning methods have driven the transition of cattle behavior recognition from manually designed features to end-to-end learning, achieving significant progress in both recognition accuracy and generalization ability.

However, most existing studies still rely primarily on visible-light images as input. In real-world large-scale farming scenarios, feature extraction from a single RGB modality is highly dependent on the shooting angle and image quality, making it highly susceptible to interference from complex background elements such as fences, feed troughs, and ground stains. Furthermore, when cattle are densely distributed, visual features between individuals are prone to overlap and confusion, leading to a decline in behavioral recognition accuracy. The pure RGB modality primarily captures the color, texture, and two-dimensional appearance of cattle, lacking an effective description of their physical spatial structure, true distance distribution, and depth morphology. This lack of spatial geometric information makes it difficult for the model to precisely define the key interaction areas and body contour boundaries for specific behaviors, which severely limits the model’s detection accuracy and robustness in complex barn environments.

Although introducing depth information offers a promising avenue for mitigating the aforementioned limitations, existing RGB-Depth livestock studies still leave several critical gaps. Lee et al. [17] employed a Kinect depth sensor to recognize aggressive behavior in pigs, demonstrating the potential of depth cues for behavior analysis; however, such approaches rely on dedicated depth cameras, which incur extra hardware costs, multi-camera calibration errors, and spatio-temporal synchronization difficulties—practical burdens that are particularly acute in large-scale, cost-sensitive farming environments. Tan et al. [18] proposed a dual-stream fusion network (ConvNeXtV2) for pig weight estimation using RGB-D data, achieving improved accuracy through two-stream feature fusion; nevertheless, their method focuses on morphological measurement rather than multi-class behavior detection, and its two-stream backbone entails considerable parameter and computational overhead.

To address the aforementioned issues, this paper proposes a method for cattle behavior detection (DMNet) based on RGB-Depth Dual-Modal Information Fusion. By introducing the depth modality to provide spatial structural priors, it complements the visual semantics of the RGB modality. The method also optimizes for noise interference, scale variability, and computational redundancy in feature fusion. The main contributions of this paper are as follows:

(1) We constructed a cattle behavior detection framework based on RGB-Depth Dual-Modal Information Fusion. This framework employs a dual-branch backbone network that utilizes the geometric prior of the depth channel to perform structural regularization, effectively compensating for the deficiencies of the single RGB modality in describing physical spatial structures, and achieving efficient complementarity and integration of appearance texture and spatial geometric information.
(2) We propose the CDSAM module to suppress complex background noise and adapt to varying poses. To address the issues of background interference in barn environments and the tendency of static convolutions to amplify noise due to the cattle’s dynamic poses, this module combines a parameter-free attention mechanism with dynamic convolutions. It adaptively evaluates the importance of neurons and generates input-specific convolutional kernels, thereby enhancing the model’s robustness to varying poses and its ability to suppress background noise.
(3) The C2BRA module was proposed to enhance multi-scale spatial context representation capabilities. To address the challenge of feature extraction caused by variations in the scale of individual cattle, we introduce a two-layer routing attention mechanism. Through a two-stage modeling approach—“region-level routing” followed by “fine-grained attention within regions”—the model adaptively identifies highly relevant regions, thereby enhancing its ability to perceive key areas of behavioral interaction across different scales and improving the quality of spatial boundary representations.
(4) A lightweight shared convolutional detection head (LSCD) was designed to reduce computational overhead. To address the issue of parameter redundancy caused by multi-scale prediction branches, the LSCD employs cross-scale shared convolutional parameters and a decoupled classification/regression architecture, reducing the model’s computational complexity while maintaining detection accuracy.

## 2. Materials and Methods

### 2.1. Materials

#### 2.1.1. Data Sources

**Figure 1:**
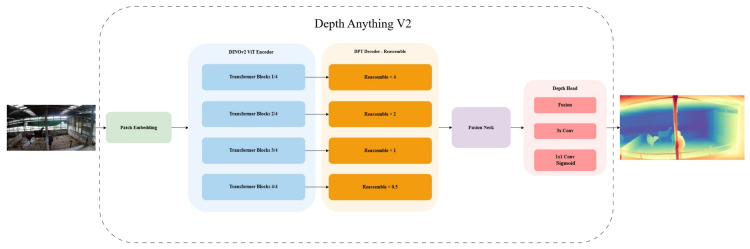
The process of generating depth images.

The cattle behavior data for this study were collected at the ranch of Chaomei Food Co., Ltd. in Luhe County, Shanwei City, Guangdong Province, China. The ranch primarily raises Simmental and Angus cattle. To ensure the naturalness and authenticity of the behavioral data, no physical restraints or human interventions were applied to the cattle during data collection. Video data were collected using high-definition cameras between November 2025 and January 2026. For depth modal acquisition, this study did not use traditional depth camera hardware but instead employed a monocular depth estimation model (Depth Anything V2) [19] to generate corresponding depth map, The generation process is shown in Figure 1.

**Figure 2:**
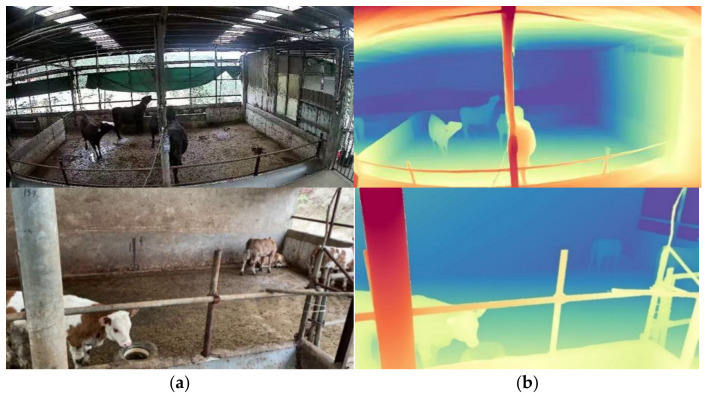
RGB and depth images of cattle behavior: (**a**) RGB images; (**b**) depth images.

Each frame of an RGB image is first divided into a sequence of image patches via Patch Embedding, and a pre-trained DINOv2 Vision Transformer encoder extracts multiscale semantic features layer by layer; subsequently, the DPT decoder performs channel projection and multiscale realignment of the features from each layer via the Reassemble module, and the Fusion Neck fuses them into a unified dense representation; finally, a relative depth map aligned with the input image at the pixel level is output by the deep prediction head. It should be noted that since this depth map is estimated from RGB images via a data-driven model, it is essentially a synthetic geometric representation derived from RGB data, rather than an actual physical measurement captured by an independent depth sensor; therefore, strictly speaking, the framework described in this paper is a pseudo-dual modal architecture based on RGB data, rather than hardware-level fusion of heterogeneous sensors. The primary considerations for adopting this generation of strategies are ease of deployment and cost advantages: there is no need to configure additional depth cameras, which fundamentally avoids calibration errors and spatiotemporal synchronization issues among multiple cameras, ensures strict pixel-level alignment between RGB images and depth maps, and significantly lowers the hardware barriers for deployment in actual livestock farms. Figure 2 shows examples of RGB images taken from different areas and viewpoints in a real cattle barn environment, along with the corresponding generated depth maps.

#### 2.1.2. Dataset Creation

**Figure 3:**
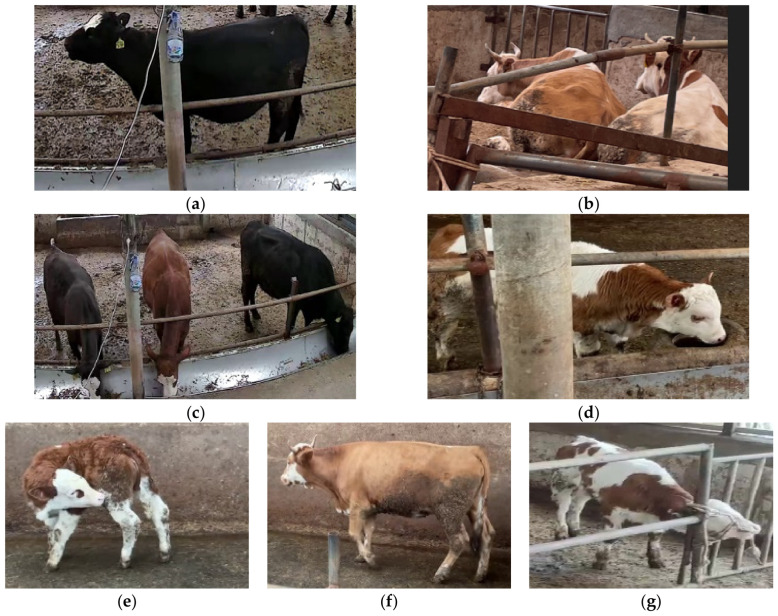
Images of different behaviors of cattle: (**a**) standing; (**b**) lying; (**c**) eating; (**d**) drinking; (**e**) licking; (**f**) walking; (**g**) searching.

In this study, cattle behaviors were classified into seven categories: standing, lying, eating, drinking, licking, walking, and searching. Among them, standing, lying and eating were the high-frequency behaviors characterizing the basic physiological state. Licking and walking enriched the fine behavioral phenotype of cattle in natural rearing environment. This classification system reflects behavioral characteristics observed in real-world farming scenarios. The detailed criteria for determining each category are shown in Table 1, and an example of typical behavior is shown in Figure 3.

During the data preprocessing stage, the collected raw video was first frame-extracted and manually screened to remove invalid samples where the target was missing, the image was blurry, or the image quality was poor. Subsequently, the LabelImg (1.8.6) tool was used to annotate the target bounding boxes and behavior categories in the RGB images, and the annotation results were synchronously mapped to the corresponding depth images, thereby achieving consistent alignment of spatial and semantic information across the dual-modal sample space.

To prevent data leakage, this study adopted a video-sequence-based partitioning strategy: using a single video file as the smallest partitioning unit, ensuring that all frames from the same video segment appear in only one of the training, validation, or test sets, with no overlap among the three sets at the video level; the dataset was split into a training set (2194 pairs), a validation set (274 pairs), and a test set (274 pairs) in an 8:1:1 ratio. To address issues such as dense and overlapping objects in the cattle barn setting, data augmentation strategies—including horizontal flipping, illumination enhancement, and motion blur—were applied to increase sample diversity and feature richness, thereby enhancing the model’s robustness in complex environments.

### 2.2. Method

#### 2.2.1. Dual-Modal Network (DMNet)

**Figure 4:**
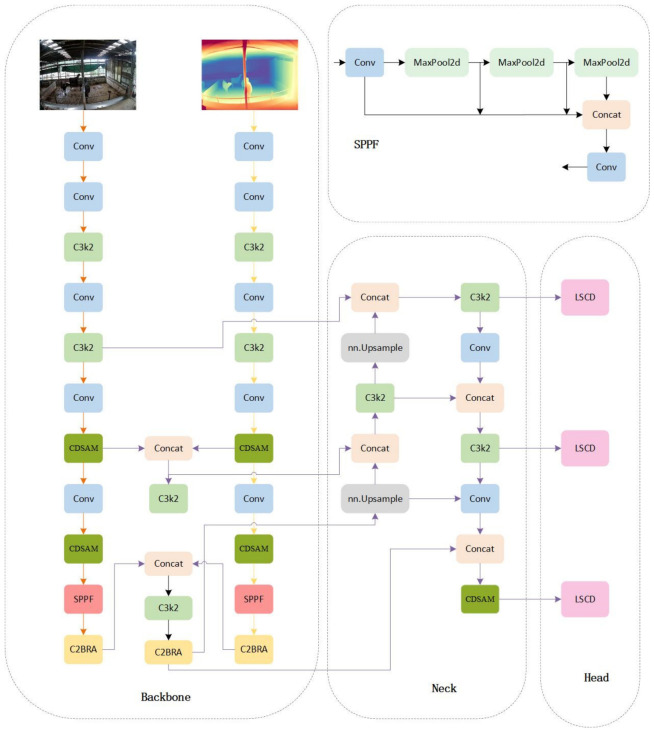
DMNet model.

Building upon recent advances in intelligent deep learning architecture design and optimization [20], this paper proposes an improved RGB-Depth dual-modal object detection model, whose overall structure is shown in Figure 4. The model consists of three main components: a backbone network, a neck network, and a detection head. This model employs two branches for parallel feature processing: the RGB stream (orange path) and the depth stream (yellow path), integrating complementary multi-scale information during a two-stage fusion process. In-depth optimization has been performed across three dimensions—feature extraction, scale representation, and detection head lightweighting—to systematically address the challenges posed by complex scene interference and the difficulty of extracting key behavioral features.

#### 2.2.2. CDSAM Module

**Figure 5:**
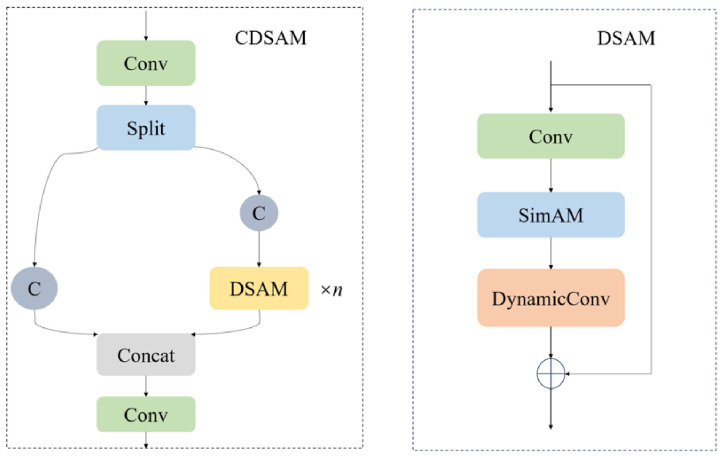
Structure of the CDSAM module.

To address the challenges of background noise and highly variable cattle poses in cattle barn scenarios—where the static convolutions of the original C3k2 module fail to adapt to feature distributions and tend to amplify noise—this paper proposes an improved version of C3k2, the CDSAM module. This module retains the cross-stage local architecture, with its core innovation being the replacement of the original static bottleneck module with DSAM, as shown in Figure 5. The DSAM module internally connects conventional convolutions, a parameter-free attention mechanism, and dynamic convolutions in series.

Rooted in neuroscience theories, SimAM evaluates the importance of each neuron within an individual channel by defining an energy function. For a specific target neuron t, its minimum energy $e_{t}^{*}$ is calculated as follows:

(1) $$e_{t}^{*}=\frac{4\left(\sigma^{2}+\lambda \right)}{{\left(t-\mu \right)}^{2}+2\sigma^{2}+2\lambda }$$

In the equation, μ and $\sigma^{2}$ represent the mean and variance of all neurons in the current feature channel, respectively, and λ is the regularization hyperparameter.

**Figure 6:**
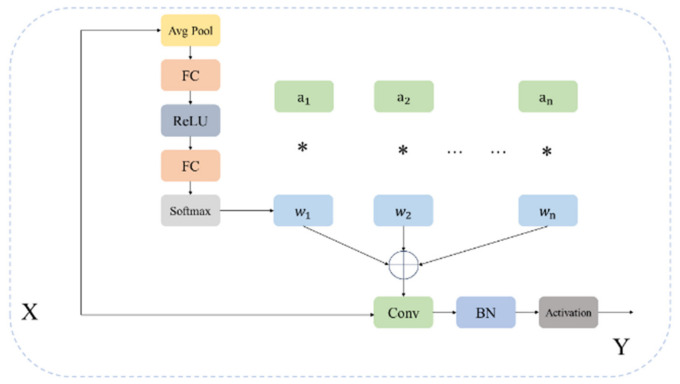
DynamicConv module. The symbol * denotes the weighted aggregation operation.

DynamicConv [21] dynamically generates convolutional kernels based on the distribution of input features to adaptively accommodate the highly variable postures of cattle, and its architectural mechanism is illustrated in Figure 6. First, the global context information of the input feature map X is aggregated via global average pooling. This aggregated feature is then mapped through a two-layer multilayer perceptron (MLP) containing a ReLU activation function, and the Softmax function is utilized to generate the dynamically normalized weights $w_{i}$ for n expert convolutional kernels. Subsequently, a linear weighting is performed on the n mutually independent expert kernels $a_{i}$ to aggregate and generate the dynamic convolutional kernel $\tilde{W}$ dedicated to the current input:

(2) $$\tilde{W}=\sum_{i=1}^{n}w_{i}\cdot a_{i}\;$$

Finally, the generated dynamic convolutional kernel $\tilde{W}$ is utilized to perform a convolutional mapping on the input feature X. The mapped feature is sequentially processed by Batch Normalization (BN) and an activation function (Activation) to yield the final output Y:

(3) $$Y=\mathrm{Activation}\left(\mathrm{BN}\left(\tilde{W}*X\right)\right)$$

#### 2.2.3. C2BRA Module

**Figure 7:**
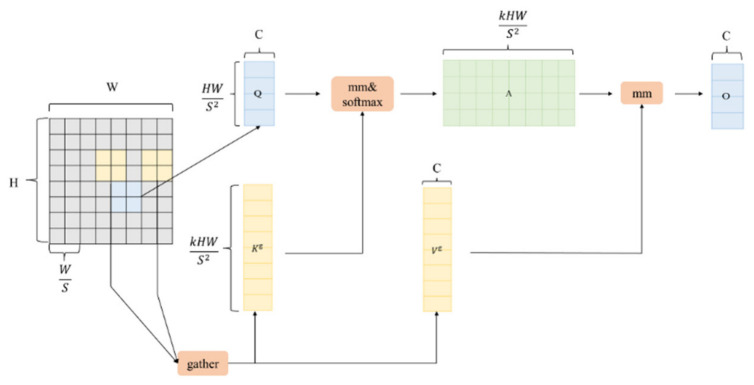
Bi-Level routing attention module.

In the task of dual-modal cattle behavior detection, individual cattle often exhibit significant variations in size due to differences in shooting distance and imaging angles. The same behavior category may correspond to different spatial scales in the feature map: larger cattle typically possess more complete information regarding trunk and limb structures, whereas smaller cattle retain only limited local response regions. When using feature modeling approaches with fixed local receptive fields, models often struggle to simultaneously capture the overall structural representation of large-sized targets and extract key information from small-sized targets, thereby affecting behavior recognition performance. To address the above issues, this paper introduces Bi-Level Routing Attention (BRA) [22] based on the C2PSA module to construct the C2BRA module, thereby enhancing the network’s ability to adaptively model target features at different scales. The basic structure of BRA is shown in Figure 7.

The core of BRA adopts a two-stage modeling approach consisting of “region-level routing and intra-region fine-grained attention”. First, the input feature map $X\in R^{H\times W\times C}$ is divided into S×S non-overlapping regions, each of size $\frac{H}{S}\times \frac{W}{S}$. In the region-level routing phase, the affinity mapping (adjacency matrix) $A^{r}$ between different regions is derived by computing the inner product of their respective regional feature vectors:

(4) $$A^{r}=Q^{r}{\left(K^{r}\right)}^{T}$$

Subsequently, a top-k routing strategy is applied to adaptively select the k most relevant candidate regions for each region. This mechanism can bridge physical spatial scales, thereby effectively filtering out meaningless and redundant background noise. During the intra-region attention phase, fine-grained, token-level feature interactions are executed exclusively within the activated k candidate regions. Assuming the query matrix of the current region is Q, and the key and value matrices derived from routing aggregation are $K^{g}$ and $V^{g}$, respectively, the core computation process of the intra-region attention is formulated as follows:

(5) $$O=\mathrm{Softmax}\left(\frac{Q{\left(K^{g}\right)}^{T}}{\sqrt{d}}\right)V^{g}$$

Compared to global self-attention mechanisms, BRA is able to precisely aggregate multi-scale semantic information most relevant to the current behavioral goal through highly dynamic receptive fields, while significantly reducing redundant computations.

**Figure 8:**
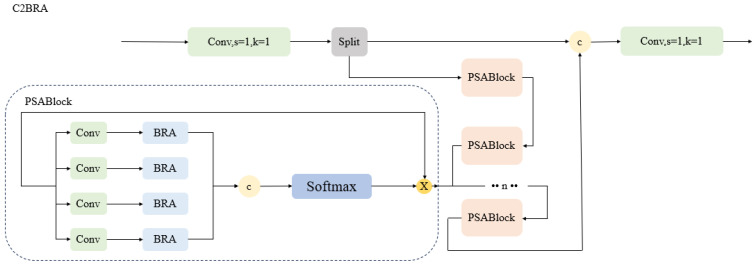
Structure of the C2BRA module.

Compared to the original C2PSA, the main improvement of C2BRA lies in replacing the attention units within the PSABlock with BRA, enabling the module to adaptively select more discriminative regional information based on changes in the target scale. Its structure is shown in Figure 8. The input features first undergo channel-wise adjustment via convolution, then are split into two parts along the channel dimension: one part is passed directly through a shortcut branch to preserve the original information, while the other enters the main branch, sequentially passing through multiple PSABlocks embedded with BRA for feature extraction and attention enhancement. Finally, the features from the main branch and the shortcut branch are concatenated along the channel dimension, and channel fusion is performed via convolution to obtain the module’s output. For large-sized objects, this approach enhances the representation of overall contours and pose structures; for small-sized objects, it highlights responses in key local regions. This module helps improve the model’s feature representation capabilities under conditions of significant individual size variation among cattle, providing more effective multi-scale semantic information for subsequent detection branches.

#### 2.2.4. LSCD Detection Head

**Figure 9:**
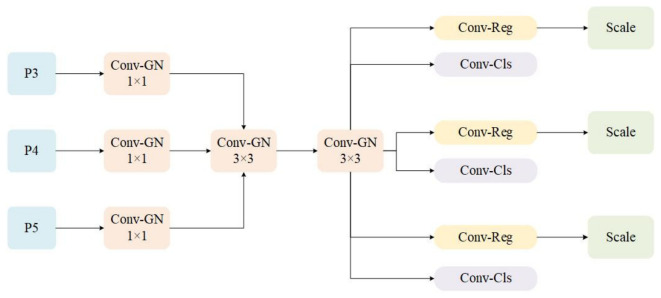
LSCD module.

In the task of dual-modal cattle behavior recognition and detection, behavioral targets such as standing, lying down, eating, and drinking exhibit significant scale variations across different scenarios. Conventional object detection heads typically construct independent prediction branches for feature streams of different scales. While this design can mitigate scale-sensitivity issues, the repetition of the base network architecture easily leads to a dramatic increase in parameters and excessive computational overhead. To address this, this study introduces a lightweight shared convolutional detection head [23], whose structure is shown in Figure 9.

The core concept of LSCD is to share convolutional parameters across multi-scale detection branches, allowing features from different scales to reuse the same set of convolutional transformations during the prediction phase, thereby significantly reducing the parameter count and computational complexity. Specifically, multi-scale features from P3, P4, and P5 first undergo channel alignment and feature normalization via individual 1×1 Conv-GN blocks, respectively, and are subsequently processed by a shared 3×3 Conv-GN module to complete unified feature transformation and reorganization. To enhance the stability of feature normalization and mitigate sensitivity to batch size, LSCD adopts Group Normalization (GN) instead of the commonly used Batch Normalization (BN) within the shared convolutions. This ensures a more stable feature distribution in multi-scale prediction scenarios, ultimately contributing to improved convergence and generalization performance throughout the detection process.

During the prediction phase, LSCD employs a decoupled design for classification and regression, where shared features are fed into the classification branch and the regression branch after entering the detection head: the classification branch outputs the confidence level of the cattle behavior category, while the regression branch predicts the parameters of the target bounding box. Although the branches at different scales share the convolutional transformation process, classification and regression utilize independent output layers to complete the final prediction, thereby reducing computational costs while maintaining the necessary predictive capability. Furthermore, the regression branch introduces a scale adjustment factor at the output of each scale to scale and correct the regression results, thereby adapting to the regression requirements of targets at different scales and reducing biases caused by scale differences. The formal representation is as follows. Let the input feature map of the l-th scale be denoted as:

(6) $$F_{l}\in R^{C\times H_{l}\times W_{l}},l\in \left\{3,4,5\right\}$$

In the equation, C is the number of channels after pooling. The shared convolution operation can be expressed as:

(7) $$G_{l}={Conv}_{shared}\left(F_{l}\right)$$

In the equation, ${Conv}_{shared}$ represents the convolutional module with parameter sharing across all scale branches. The outputs are then obtained through the classification and regression prediction layers, respectively:

(8) $$P_{l}^{cls}={Conv}_{cls}\left(G_{l}\right),P_{l}^{reg}={Conv}_{reg}\left(G_{l}\right)$$

In the equation, ${Conv}_{cls}$ and ${Conv}_{reg}$ represent the classification and regression prediction convolutions, respectively. The regression branch scales the prediction at the scale end to meet the regression requirements for targets of different scales.

In summary, LSCD effectively reduces parameter redundancy and computational overhead in multi-scale detection heads through cross-scale shared convolutions and a stable normalization strategy. By combining classification/regression decoupling with a scale adjustment mechanism, it enhances the model’s deployability and inference efficiency in dual-modal cattle behavior detection tasks while maintaining detection accuracy.

## 3. Results

### 3.1. Test Platform and Parameter Settings

In this study, the image input size was set to 640 × 640. Training was performed using the stochastic gradient descent (SGD) algorithm with 300 training epochs, a batch size of 32, and 4 worker threads. The specific experimental environment configuration is shown in Table 2.

### 3.2. Evaluation Criteria

To comprehensively evaluate the performance of the proposed model in the task of cattle behavior detection, this paper adopts mean average precision (mAP), a commonly used metric in the field of object detection, as the primary performance evaluation metric. Additionally, to comprehensively assess the model’s scale and computational complexity, the number of model parameters and computational cost (GFLOPs) are introduced as auxiliary evaluation metrics.

mAP is a key metric for evaluating the overall performance of object detection models, as it reflects the model’s detection accuracy across different recall levels. It is defined as the arithmetic mean of the average precision (AP) across all classes, calculated as follows:

(9) $$mAP=\frac{1}{C}\sum_{i=1}^{C}{AP}_{i}\times 100\%\;$$

where C denotes the number of detection categories and ${\mathrm{AP}}_{i}$ represents the average precision of the i-th target class.

Precision and recall are used to measure the accuracy and completeness of detection results, respectively, and are defined as follows:

(10) $$P=\frac{TP}{TP+FP}$$

(11) $$R=\frac{TP}{TP+FN}$$

Here, TP (True Positives) refers to the number of targets correctly detected; FP (False Positives) refers to the number of objects incorrectly identified as targets; and FN (False Negatives) refers to the number of actual targets that were not detected. Precision reflects the reliability of the model’s predictions, while recall measures the model’s ability to capture actual targets. mAP takes into account how both metrics vary across different thresholds and is the most representative evaluation metric for object detection tasks.

The number of model parameters is used to measure the size of a model, reflecting its storage requirements and the potential risk of overfitting. For convolutional neural networks, the number of parameters primarily comes from the learnable weights in each layer, and the total number of parameters can be expressed as:

(12) $$\mathrm{Parameters}=\sum_{l=1}^{L}\left(C_{\mathrm{out}}^{\left(l\right)}\times C_{\mathrm{in}}^{\left(l\right)}\times K_{l}\times K_{l}\right)$$

where L denotes the total number of network layers, $C_{\mathrm{in}}^{\left(l\right)}$ and $C_{\mathrm{out}}^{\left(l\right)}$ represent the number of input and output channels of the l-th layer, respectively, and $K_{l}$ is the convolutional kernel size of that layer.

Computational complexity is evaluated in terms of floating-point operations (FLOPs). In this paper, GFLOPs ($10^{9}$ floating-point operations) are adopted as the metric unit to evaluate the computational overhead of the model during the forward inference phase. For a convolutional layer, its FLOPs can be formulated as:

(13) $$\mathrm{LOPs}=H\times W\times C_{\mathrm{out}}\times C_{\mathrm{in}}\times K\times K\;$$

where H and W represent the spatial dimensions of the output feature map. The total GFLOPs of the model are obtained by aggregating the FLOPs across all individual layers.

#### Comparative Evaluation of Different Object Detection Algorithms

**Figure 10:**
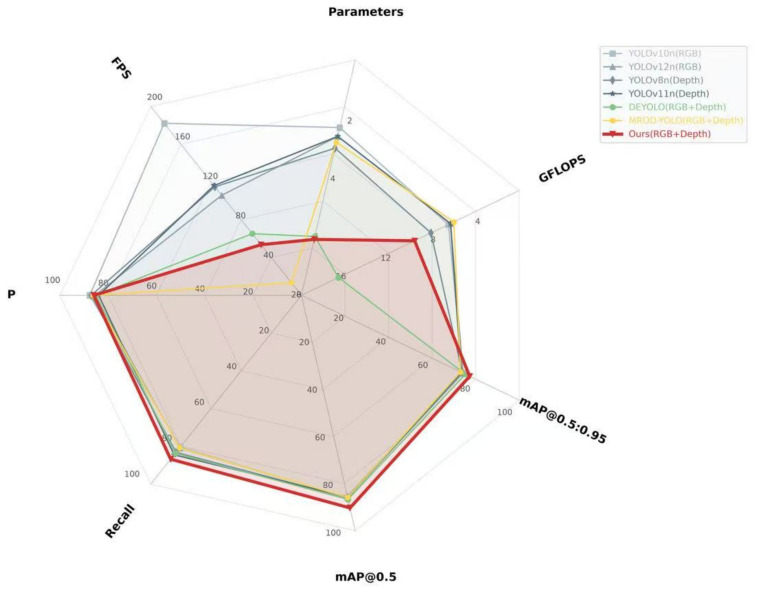
Radar charts of some detection models.

To validate the effectiveness of the proposed method in the task of cattle behavior detection, this paper selects the two-stage detectors SSD and Faster R-CNN, the single-stage YOLO series models [24,25,26,27], the Transformer-based RT-DETR [28], and several multimodal algorithms [29,30,31] as comparison methods, and conducts experiments under three input configurations: RGB, Depth, and RGB+Depth. The results of the comparative experiments for each model are shown in Table 3. Figure 10 shows a radar chart of the metrics for some of the models.

Under monomodal input conditions, the YOLO series of models, leveraging their lightweight single-stage fully convolutional architecture, outperformed both two-stage detectors and Transformer-based models in overall detection performance, demonstrating their strong applicability in complex cattle barn environments. In the RGB modality: YOLOv10n achieved the highest precision among single-modality models, reaching 87.6%; while YOLOv12n performed best in terms of mean average precision (mAP@0.5:0.95), reaching 74.2%. In the depth modality: YOLOv8n achieved a precision of 87.0%, and YOLOv11n achieved a recall of 84.5%. These results indicate that the spatial structure and 3D geometric information contained in the depth channel can effectively compensate for the lack of RGB visual information in specific scenarios, enhancing the feature representation of the cow’s 3D silhouette. However, in terms of overall metrics, the average accuracy of most models in the single depth modality is slightly lower than that in the RGB modality, confirming that relying solely on depth information makes it difficult to fully characterize the subtle appearance textures and fine-grained semantic differences in cattle behavior.

However, from the perspective of cross-channel fusion, simple multimodal stacking does not necessarily lead to a linear performance leap. As shown in Table 2, after introducing dual-modal inputs, existing dual-modal comparison models exhibited different convergence characteristics: although DEYOLO and UMIS-YOLO outperform most single-modal baselines across all accuracy metrics, both exhibit varying degrees of expansion in parameter count and computational complexity. Notably, UMIS-YOLO’s computational cost surges to 98.1 G, severely limiting its ability for real-time deployment on edge devices. Although MROD-YOLO demonstrates excellent lightweight characteristics, its detection capability is limited due to insufficient interaction between heterogeneous modal features during extraction, resulting in a mAP@0.5 of only 85.8%, which is even lower than some excellent single-modal baselines.

Compared to all single-modal and similar dual-modal comparison models, the dual-modal model proposed in this study demonstrates the best detection performance. Its precision, recall, mAP@0.5, and mAP@0.5:0.95 reach 85.9%, 86.8%, 90.3%, and 77.3%, respectively. Among these, the recall and both metrics achieve the highest values. Compared to existing dual-modal models, the proposed model achieves a significant improvement of over 3.5% in mAP@0.5 and over 2.0% in mAP@0.5:0.95. The experimental results demonstrate that, compared to traditional heterogeneous modality fusion strategies, the feature alignment and cross-stage dynamic parameter-free attention mechanisms designed in this study can more efficiently activate the appearance and texture of RGB and the spatial geometric manifold of depth, achieving true complementary advantages between the two modalities.

In terms of model complexity, the proposed method has 6.1 million parameters, a computational cost of 9.6 GFLOPs, and an inference speed of 53.6 FPS. Although its complexity is slightly higher than that of lightweight models such as YOLOv10n, YOLOv11n, and YOLOv12n, it is significantly lower than that of comparison models such as SSD, Faster R-CNN, and RT-DETR, while achieving superior results in accuracy metrics. Notably, despite employing two parallel backbones, DMNet still achieves a real-time inference speed of 53.6 FPS. This indicates that our method maintains good lightweight characteristics and real-time deployment capability while improving detection performance, striking a reasonable balance between detection accuracy, computational complexity, and inference efficiency.

To further validate the detection performance of our method across different cattle behavior categories, we calculated the detection accuracy of each model for the seven behavior classes, with the results shown in Table 4.

As shown in Table 4, there are significant differences in detection performance across various cattle behaviors among the different models. Compared to other models, the RGB+Depth dual-modal method proposed in this paper demonstrates the best overall performance, achieving an mAP@0.5 of 90.3%. Looking at specific categories, our method achieved the highest detection accuracy for all five behavior categories—walking, standing, lying, licking, and searching—reaching 62.6%, 91.7%, 99.4%, 98.2%, and 92.7%, respectively.

A further analysis reveals that the spatial-depth prior yields a substantially larger performance gain for dynamic behaviors than for static behaviors. This discrepancy stems from the inherent difference in the spatio-temporal characteristics of the two behavior types. Lying is a highly stable behavior with a consistent two-dimensional appearance: the cattle body is largely pressed against the ground with minimal deformation, so the RGB texture alone already provides sufficiently discriminative features, leaving only limited marginal gain for the depth prior. Walking, by contrast, involves continuous limb displacement, gait transitions, and changes in relative position between individuals, making it difficult to reliably delineate the body boundary and spatial location of a moving animal from RGB appearance alone. The geometric-structure cues provided by the depth prior assist the model in separating the moving subject from the background in the spatial dimension, thereby alleviating the boundary blurring caused by displacement and local occlusion. Consequently, the improvement on walking is markedly more pronounced than on lying, indicating that the spatial-depth prior provides stronger structural regularization for dynamic tasks characterized by intense spatio-temporal variation and unstable appearance cues than for the discrimination of static postural states.

Overall, our method achieved superior results across most behavior categories, indicating that the visual information in RGB images and the spatial structural information in depth images are highly complementary. This synergy effectively improves the accuracy and stability of multi-behavior detection in complex cattle barn environments.

### 3.3. Ablation Experiments

To evaluate the impact of the three improved modules—CDSAM, C2BRA, and LSCD—on the performance of cattle behavior detection, we conducted ablation experiments on the baseline model. The results are shown in Table 5.

As shown in Table 5, all three modules—CDSAM, C2BRA, and LSCD—improve the performance of cattle behavior detection, but their contributions differ. Compared to the baseline model, introducing only CDSAM increased recall from 80.9% to 85.3%, a 4.4 percentage point improvement; mAP@0.5 rose from 86.0% to 89.4%, an increase of 3.4 percentage points; mAP@0.5:0.95 rose from 75.3% to 76.8%, an increase of 1.5 percentage points, indicating that this module enhances the network’s ability to adaptively extract features and suppress noise in the face of the highly variable shapes of cattle in complex barn environments. After introducing only C2BRA, recall, mAP@0.5, and mAP@0.5:0.95 increased by 3.9, 2.1, and 0.3 percentage points, respectively, indicating that this module possesses stronger multi-scale spatial context representation capabilities, which helps to more accurately capture cow behavior regions with significant multi-scale heterogeneity. Upon introducing LSCD alone, mAP@0.5 improved by 1.9 percentage points, while GFLOPs decreased from 11.0 to 10.3 (a reduction of 0.7), and the number of parameters decreased from 5.0 M to 4.8 M (a reduction of 0.2 M). This indicates that LSCD maintains good prediction performance while reducing head detection redundancy.

The combined results show that the three modules complement each other well. When all three modules are introduced simultaneously, the model achieves optimal overall performance, with recall, mAP@0.5, and mAP@0.5:0.95 reaching 86.8%, 90.3%, and 77.3%, respectively—improvements of 5.9, 4.3, and 2.0 percentage points over the baseline model. while GFLOPs decreased from 11.0 to 9.6, a reduction of 1.4. The results indicate that the collaboration of these three modules can more effectively improve the accuracy and efficiency of cattle behavior detection in complex barn environments.

To verify the stability and statistical significance of the reported performance, we repeated the training of the proposed DMNet using six different random seeds, as shown in Table 6. The mAP@0.5 value ranged from 89.5% to 90.4%, with an average of 90.08% ± 0.32%. The relatively low standard deviation indicates that the performance improvement of the proposed method is consistent and reliable.

### 3.4. Visual Analysis

**Figure 11:**
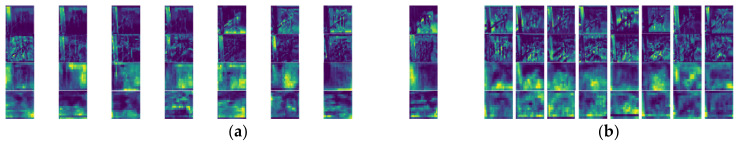
Feature maps before and after replacing the CDSAM module: (**a**) shows the feature map before replacing the CDSAM module; (**b**) shows the feature map after replacing the CDSAM module.

Figure 11 shows a comparison of multiscale feature maps before and after adding the CDSAM module in the cattle behavior detection task. Darker colors indicate low response, while yellow to green regions indicate high activation response. The comparison reveals that replacing the original C3k2 module with the CDSAM module results in a more concentrated and coherent spatial distribution of feature responses, reducing interference from complex backgrounds and irrelevant textures in the cattle barn, such as fence obstructions and floor stains. The improved CDSAM module enables the model to focus more adaptively on the cattle’s varied postures, extracting feature representations with higher discriminative power and clearer structure, thereby effectively improving the accuracy of subsequent behavior classification and regression prediction.

**Figure 12:**
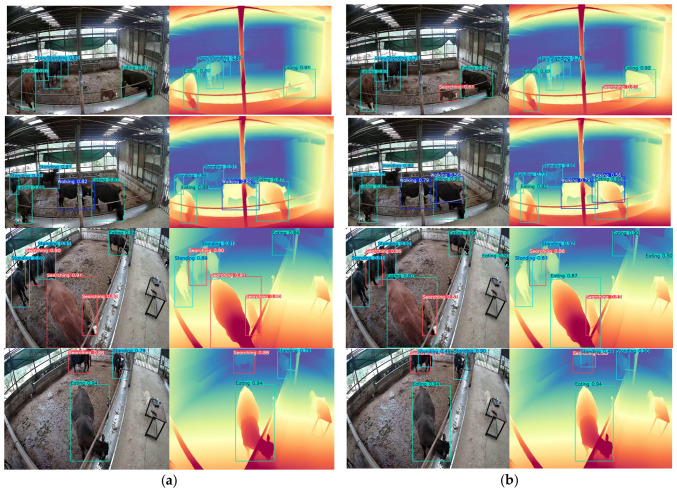
Visual comparison of results from the dual-modal detection model. (**a**) represents the baseline model; (**b**) represents the improved model.

Figure 12 shows the detection results of the model before and after improvement in real-world farming scenarios. It can be observed that when dealing with different shooting angles and activity areas—especially in complex situations such as object occlusion, close proximity, or targets located at the far end of the frame—the improved model demonstrates advantages over the baseline model: it enhances the ability to detect small-scale, weak targets and complex regions with local deformations, achieves more complete individual separation, and shows no obvious missed detections or class confusion.

## 4. Discussion

The superiority of multimodal fusion over monomodal methods has gained increasing recognition in precision livestock visual tasks. Recent studies have shown that combining RGB with modalities such as depth, thermal infrared, or multispectral data can effectively mitigate visual confusion caused by complex environments and enhance the model’s ability to represent the spatial boundaries of specific behaviors. Based on this consensus, the model presented in this paper further advances multimodal fusion for cattle behavior, moving beyond traditional simple concatenation.

To further clarify the advantages and positioning of the proposed method, we compare it with representative animal-behavior-analysis approaches. Khin et al. [32] proposed a posture-analysis-based predictive model for cattle calving time that identifies key postures such as “sitting with leg extended” (SLE) to distinguish normal from abnormal calving events, demonstrating the effectiveness of fine-grained posture modeling in livestock behavior analysis. However, that method primarily relies on two-dimensional posture features extracted from single-viewpoint RGB images, making it sensitive to complex backgrounds and illumination changes, and it lacks an explicit characterization of the spatial-geometric context in which behaviors occur. By contrast, the proposed method introduces a depth modality that provides spatial-structure priors, effectively suppressing interference from complex barn backgrounds while preserving a lightweight architecture, and enhancing the representation of the spatial boundaries of dynamic behaviors such as walking. This comparison indicates that, relative to a purely appearance-driven posture-analysis paradigm, RGB-Depth dual-modal fusion offers stronger robustness and broader applicability for multi-class behavior detection in complex environments.

Although the model in this paper achieves high overall detection accuracy, the study still has the following limitations: first, class imbalance is inevitable in the dataset, particularly with relatively limited samples of “walking” and certain fine-grained actions. This non-uniform distribution essentially reflects the real-world temporal patterns of beef cattle behavior in daily barn environments. The geometric priors of the depth channel serve as structural regularization under such data, mitigating to some extent the overfitting tendency that small-sample RGB images often cause in single-stage networks, the insufficiency of samples still limits the optimal representation of features across all categories. Second, since the depth maps employed here are synthesized by a monocular model from RGB images rather than captured by physical sensors, the depth channel constitutes an RGB-derived synthetic modality. Although this property offers significant deployment convenience, it also implies that the information content of the depth channel is bounded by the representational capacity of the depth estimation model itself, and it cannot compensate for the inherent information loss of RGB images under extreme illumination or severe occlusion.

Furthermore, since all data were collected from a single farm, the generalization of the proposed model to other farms, illumination conditions, and management systems has not yet been validated. It should be acknowledged that factors such as differing barn layouts, lighting variations (e.g., natural daylight versus artificial illumination) and breed-specific morphology may introduce domain shifts that could affect detection performance. Several design choices of DMNet are expected to confer a degree of cross-domain robustness: the CDSAM module’s dynamic convolution adapts to varying feature distributions, which helps accommodate posture and appearance differences across breeds; the depth prior generated by Depth Anything V2 provides a modality-agnostic geometric regularization that is less sensitive to illumination changes than RGB appearance; and the C2BRA module’s region-level routing adaptively selects discriminative regions across scales, which may alleviate viewpoint-induced variations. A rigorous, multi-site validation is nevertheless required to quantify this generalization, and is planned as a priority direction of future work.

To address these limitations, future research will proceed in the following directions: first, we will continue to expand the sample size of different behavioral categories to improve the balance of features across all categories and enhance the model’s generalization ability; second, we will introduce temporal feature streams to leverage the temporal continuity between video frames, thereby overcoming the limitations of single-frame optical feature degradation and further improving detection stability in challenging scenarios; third, we will explore the integration of sensor modalities such as thermal infrared or night vision imaging to comprehensively evaluate and enhance the system’s robustness in behavior monitoring under all-weather conditions (especially in low-light or no-light environments), thereby deepening its application value in comprehensive animal welfare early warning systems. In addition, to verify the cross-domain generalization of the proposed framework, we plan to collect data from multiple farms encompassing different cattle breeds, diverse barn configurations, varying camera positions and illumination conditions, and distinct management practices. Transfer-learning and domain-adaptation strategies will also be explored to reduce the data and annotation requirements when deploying DMNet to new farm environments.

## 5. Conclusions

Based on the baseline architecture, we innovatively designed the DMNet framework, an RGB-Depth dual-modality detection network tailored for cattle behavior monitoring in complex barn environments. This framework employs an RGB-Depth dual-branch backbone coupled with a deep fusion architecture, leveraging depth maps as a data-driven geometric regularizer to achieve efficient cross-modality regularization rather than traditional simple feature concatenation. Within this framework, three collaborative components are seamlessly integrated to drive the performance leap. Specifically, we developed the CDSAM module to adaptively accommodate variable cattle postures and suppress amplified background noise, thereby enhancing the ability to extract behavioral features in challenging scenarios. Concurrently, the newly proposed C2BRA module strengthens the network’s capacity to perceive key interaction areas across diverse target scales, improving the representation quality of spatial and behavioral boundaries at the multimodal fusion layer. Finally, the traditional detection head was replaced with the lightweight shared convolutional detection head (LSCD), which effectively eliminates architectural redundancy across multi-branch detection heads, enabling the model to maintain superior accuracy while trimming down the overall computational footprint.

Experimental results demonstrate that on the self-built cattle behavior dataset, the precision, recall, and mAP@0.5 of the improved model reach 85.9%, 86.8%, and 90.3%, respectively. Notably, the mAP@0.5 represents a 4.3 percentage point increase over the baseline model. Meanwhile, the computational complexity in terms of GFLOPs drops from 11.0 to 9.6, a reduction of 12.7%. A series of systematic evaluations, ablation experiments, and visualization results demonstrate that this targeted structural innovation enables the model to strike a balance between a lightweight architecture and high accuracy in terms of overall performance, effectively reducing false positives and false negatives under conditions of partial occlusion and scale variations. In summary, DMNet offers a novel approach to multimodal perception in precision livestock farming. Future research will focus on validating this framework across a broader range of behavioral categories and data acquisition conditions, while actively advancing its embedded deployment and expansion to additional modalities to further enhance the method’s practical value in real-world production settings.

## Figures and Tables

**Table 1:** Criteria for judging the cattle behavior.

Category of Behavior	Behavior Description
Standing	Standing on all fours with no specific head movements
Lying	With limbs bent and the entire abdomen pressed against the floor
Eating	While standing, the cattle reach into the feed trough or feeding stall
Drinking	While standing, the cattle lower their heads or bend them down to drink from the trough
Licking	As the cattle turn their heads, they lick their bellies
Walking	The hooves are raised at different heights, the legs are crossed, and the head is not touching the ground
Searching	The cattle’s heads are close to the ground or touching it; the cattle’s heads are close to a wall or railing; the cattle raise their heads or look around

**Table 2:** Test environment.

Hardware Specifications	Parameters
CPU	AMD EPYC 7742 64-Core Processor
RAM	128
GPU	A100-SXM4-40GB
CUDA	CUDA version: 12.4
Language	Python 3.10
Development Environment	Visual Studio Code 1.98.2

**Table 3:** Comparative test of different detection models.

Modal	Models	P	Recall	mAP@0.5	mAP@0.5:0.95	GFLOPS	Parameters/M	FPS
RGB	SSD	82.8	71.8	79.6	56.5	61.4	24.4	144.9
	Faster-RCNN	73.9	86.6	84.0	58.0	370.2	137.1	27.9
	YOLOv6n	84.0	83.9	85.8	73.8	11.8	4.2	118.5
	YOLOv8n	82.6	83.6	85.2	72.7	8.1	3.0	91.9
	YOLOv10n	87.6	80.4	86.0	74.1	6.5	2.3	182.2
	YOLOv11n	84.5	82.2	85.0	73.2	6.3	2.6	107.2
	YOLOv12n	85.4	80.9	85.8	74.2	6.3	2.6	105.9
	RTDETR	83.9	81.5	79.5	69.5	57.2	19.9	76.1
Depth	SSD	78.6	67.1	74.8	52.2	61.4	24.4	169.8
	Faster-RCNN	68.6	79.7	80.2	54.1	370.2	137.1	25.1
	YOLOv6n	83.6	81.5	84.0	72.2	11.8	4.2	123.2
	YOLOv8n	87.0	83.2	86.0	73.9	8.1	3.0	114.9
	YOLOv10n	83.0	81.6	84.1	72.4	6.5	2.3	127.4
	YOLOv11n	83.9	84.5	85.6	73.4	6.3	2.6	116.2
	YOLOv12n	84.6	82.5	85.1	73.3	6.3	2.6	108.2
	RTDETR	86.2	79.6	78.9	68.1	57.2	19.9	98.0
RGB+Depth	DEYOLO	85.0	83.7	86.7	75.3	16.6	6.0	65.3
	UMIS-YOLO	86.5	83.8	86.8	75.1	98.1	31.1	17.5
	MROD-YOLO	86.2	80.8	85.8	73.1	6.0	2.8	13.4
	Ours	85.9	86.8	90.3	77.3	9.6	6.1	53.6

**Table 4:** The detection accuracy of different behaviors of cattle.

Modal	Models	mAP@0.5	Walking	Standing	Lying	Eating	Drinking	Licking	Searching
RGB	SSD	79.6	50.4	85.2	89.5	89.4	85.9	75.3	81.6
	Faster-RCNN	84.0	55.4	90.2	95.7	91.1	87.1	81.7	87.0
	YOLOv6n	85.8	47.8	87.4	97.4	94.2	94.8	92.3	87.0
	YOLOv8n	85.3	45.2	87.3	96.5	95.7	94.3	92.1	85.7
	YOLOv10n	86.0	47.8	88.5	96.8	95.8	89.2	94.6	89.0
	YOLOv11n	85.0	43.8	86.4	96.8	95.1	92.1	94.3	86.2
	YOLOv12n	85.7	45.4	87.2	97.0	95.0	94.8	92.3	88.5
	RTDETR	79.5	30.1	80.5	97.2	92.6	90.7	86.2	79.4
Depth	SSD	74.8	40.6	83.4	83.4	86.7	83.2	68.1	78.2
	Faster-RCNN	80.2	47.9	88.7	88.7	86.1	84.5	80.3	85.3
	YOLOv6n	84.0	44.2	86.1	95.8	95.2	91.7	88.2	86.9
	YOLOv8n	85.9	50.9	88.8	96.9	95.0	94.9	88.3	86.8
	YOLOv10n	84.1	43.6	86.2	97.1	94.7	92.2	90.8	84.4
	YOLOv11n	85.6	48.6	86.9	96.4	94.5	93.9	92.9	86.3
	YOLOv12n	85.1	46.5	86.7	96.2	95.3	93.2	91.5	86.4
	RTDETR	78.9	29.6	81.5	96.9	93.3	89.5	84.1	77.2
RGB+Depth	DEYOLO	86.7	50.1	88.5	97.2	95.6	93.7	93.1	88.5
	UMIS-YOLO	86.8	48.5	86.2	96.9	95.4	97.0	94.9	88.5
	MROD-YOLO	85.8	48.7	86.5	96.5	96.2	94.9	89.5	88.4
	Ours	90.3	62.6	91.7	99.4	94.1	93.4	98.2	92.7

**Table 5:** Results of ablation experiment. The symbol √ indicates that this module should be added; the symbol × indicates that this module should not be added.

No.	CDSAM	C2BRA	LSCD	P	Recall	mAP@0.5	mAP@0.5:0.95	GFLOPS	Parameters
1	×	×	×	85.9	80.9	86.0	75.3	11.0	5.0
2	√	×	×	86.2	85.3	89.4	76.8	10.5	6.2
3	×	√	×	86.0	84.8	88.1	75.6	11.0	5.0
4	×	×	√	86.4	83.3	87.9	75.4	10.3	4.8
5	√	√	×	89.0	84.0	90.0	77.2	10.4	6.2
6	√	×	√	86.0	86.0	89.6	77.0	9.7	6.0
7	×	√	√	86.0	85.5	89.0	76.7	10.2	4.9
8	√	√	√	85.9	86.8	90.3	77.3	9.6	6.1

**Table 6:** Repeatability experiment.

	Seed 0	Seed 1	Seed 2	Seed 3	Seed 4	Seed 5	Baseline	Mean Std
mAP@0.5	90.3	90.0	90.2	90.1	90.4	89.5	86.0	90.08 ± 0.32

## Data Availability

Due to commercial confidentiality and non-disclosure agreements with the partner farms, the behavioral video and image data of the cattle in this study are not stored in public databases. The relevant data may be obtained from the corresponding author upon reasonable request.
